# Retraction of: Long non-coding RNA NNT-AS1 promotes cholangiocarcinoma cells proliferation and epithelial-to-mesenchymal transition through down-regulating miR-203

**DOI:** 10.18632/aging.206322

**Published:** 2025-09-30

**Authors:** Yulei Gu, Zhiqiang Zhu, Hui Pei, Dong Xu, Yumin Jiang, Luanluan Zhang, Lili Xiao

**Affiliations:** 1 Emergency Intensive Care Unit, The First Affiliated Hospital of Zhengzhou University, Zhengzhou 450052, China; 2 Department of Cardiology, The First Affiliated Hospital of Zhengzhou University, Zhengzhou 450052, China

**Keywords:** long non-coding RNA NNT-AS1, cholangiocarcinoma, proliferation, epithelial-to-mesenchymal transition, miR-203

**This article has been retracted:** This decision follows an investigation by Aging journal regarding concerns raised on PubPeer. Our image forensics analysis revealed that multiple Western blot images within the paper have been manipulated and duplicated. Specifically,

Figure 2C and 2F: images corresponding to CCLP1 and TFK1 cell lines data for cyclin D1, pro-caspase-3, and cleaved-caspase-3 appear duplicated inside panel F and between panels C and F.

Figure 3: duplicated areas are noted for CCLP1 (panel 3A) and TFK1 (panel 3C) data related to E-cadherin, N-cadherin, vimentin, Snail, and Slug.

Figure 5: shows duplicated images for CCLP1 (panel 5E) and TFK1 (panel 5G) data for cyclin D1, E-cadherin, N-cadherin, vimentin, Snail, and Slug. In addition, some western blots appear duplicated in other figures from external sources. Thus, Western blot in panel 5E overlaps with blot in Figure 2F in unrelated paper [1], with Figure 2G in [2], western blot in Figure 5C was found in paper [3], and images in Figure 5G were also present in papers [4] and [5].

Figure 6: duplicated areas are present in the images for CCLP1 (panel 6A, 6E) and TFK1 (panels 6C, 6G) cell lines data related to t-PI3K, p-PI3K, t-AKT, p-AKT, t-ERK1/2, and p-ERK1/2.

In light of all these facts the Editorial decision was made to retract this paper. The Scientific Integrity office at Aging attempted to contact the authors regarding this retraction, but has not received a response.
